# A Mouse Model for Studying the Development of Apical Periodontitis with Age

**DOI:** 10.3390/cells10030671

**Published:** 2021-03-17

**Authors:** Elisheva Goldman, Eli Reich, Bar Roshihotzki, Maya Saketkhou, Sharon Wald, Ayana Goldstein, Yehuda Klein, Itzhak Abramovitz, Michael Klutstein

**Affiliations:** 1 Faculty of Dental Medicine, Institute of Dental Sciences, The Hebrew University, Jerusalem 9112001, Israel; elishevag@ekmd.huji.ac.il (E.G.); reich.eli@gmail.com (E.R.); baroshihotzki@gmail.com (B.R.); msaketkhou@gmail.com (M.S.); Waldsharon@gmail.com (S.W.); Yehuda.klein@mail.huji.ac.il (Y.K.); 2 Hadassah Medical Center, Department of Endodontics, Jerusalem 9112001, Israel; ayanago@gmail.com

**Keywords:** apical periodontitis, immune system, aging

## Abstract

Older age is associated with reduced immune function. Our aim was to study how age affects the development of apical periodontitis (AP). AP was induced in two age groups of mice (young vs. adult). Histological samples were stained by Hematoxylin Eosin, Brown and Brenn, and Tartrate-Resistant Acid Phosphatase. In addition, the samples were scanned by Micro-Computerized-Tomography (micro-CT) to evaluate apical constriction and periapical lesion size. Cell density in the periapical region was computationally assessed. Moreover, lesion immune cell populations were characterized by flow cytometry and immunofluorescence. The young group presented more canals with necrotic radicular pulp compared to the adults. There was no difference in bacteria location in the canals between the groups. Apical constriction size was larger in the young mice compared to the adults. The periapical cell density was higher in the young group, while the dominant immune cells in the lesions were neutrophils, which also exhibited the highest young/adult ratio. Immunofluorescence demonstrated neutrophils in the lesion. More osteoclasts were present in the lesions of the young mice, in correlation to the higher volume of bone resorption in this group. Overall, we conclude that the immune reaction to AP stimuli was attenuated in the adult mice compared to the young.

## 1. Introduction

Apical periodontitis (AP) is an inflammatory lesion in the periodontal tissues surrounding the root apices. This disease is quite common, with a prevalence of 6.3% of all teeth [1]. The pathogenesis of AP following tooth necrosis originates from the associated infection which causes necrosis in the tooth pulp. The bacteria or their products (commonly Lipopolysaccharide, LPS) egress into the periapex and stimulate a complex immune response of the host. The microbial factors and host defense forces destroy the periapical tissues and form AP. Some of the cellular elements involved in AP include polymorphonuclear cells (PMN), lymphocytes, plasma cells, macrophages, and osteoclasts. Important cytokines include IL-1, 6,8, TNFα/β, and IFNα/β. Matrix metalloproteinases (MMPs) are involved in tissue degradation. It is accepted to divide the immune reaction in AP to the acute initial phase and the chronic phase. During the initial stage, pro-inflammatory cytokines are secreted and bone resorption is at its peak. During the chronic phase, the pro-inflammatory cytokines are downregulated, reducing bone resorption and the tissue reaches an equilibrium. However, this equilibrium can be disturbed, and the progression of the disease occurs in leaps, with periods of stability in-between. Since the microbes are located within the root canal, which is beyond the reach of the body’s defenses, AP is not self-healing [2]. Due to the increase in human lifespan, elderly patients are frequently seen at the dental clinic, although not much is known about how age effects the development of AP. Our hypothesis was that since AP is a disease with a strong involvement of the immune system, age-related immune changes may affect the progression of AP.

Immune-system aging, termed immunosenescence, is a complex process involving all arms of the immune system and many different cell types. In the innate immune system, older people present a reduction in cytokine signaling, production of peroxide and nitric oxide, and the phagocytic function of neutrophils. Human natural killer (NK) cells exhibit reduced activity (cytokine secretion and cytotoxicity) as well as diminished migration, and the ability of macrophages to phagocytose apoptotic cells is diminished. The uptake of antigens by DCs is reduced, decreasing consequent T cell stimulation. The adaptive immune system is substantially affected by aging and presents a decrease in the variance of the T cell receptors (TCRs), diminished levels of naïve T and B lymphocytes, and a decrease in antibody avidity in older people [3].

Another related process which occurs with old age is inflamm-aging, a chronic persistent low-grade inflammation with elevated levels of cytokines such as C-Reactive Protein (CRP), IL-6, IL-1β, and TNF. These two processes (immunosenescence and inflamm-aging) lead, on the one hand, to immune stimulation at the basal level, and on the other hand, to a decreased ability of the immune system to preform specific roles. Inflamm-aging and immunosenescence progress in parallel and form a vicious cycle. Increased production of inflammatory mediators characteristic of inflamm-aging contributes to decreases in the adaptive immune response and to immunosenescence. In contrast, the decrease in the adaptive immune response reinforces the stimulation of the innate immune response, leading to inflamm-aging. The outcome of this situation is an increased frequency of infections, cancer, and chronic diseases in the elderly [4].

Although not much work has previously been done on the progression of AP with age, a previous study on humans observed lower levels of macrophages in apical lesions in adult compared to young patients [5]. The aim of the current study was to investigate how age affects the progression of AP in a mouse model. For this purpose, we chose to use a mouse model of induced apical periodontitis for our investigation [6,7,8] and compared different age groups with the same genetic and environmental conditions.

In our study we followed the histopathological sequence of AP progression starting from root canal infection (% of necrotic canals, bacteria localization, and apical foramen volume), and ending in formation of a periapical (PA) lesion (cellular density and cellular composition of the lesion), with subsequent bone resorption (osteoclast numbers and lesion volume) [2]. 

A better understanding of how age impacts AP progression may produce information to help provide an endodontic treatment plan more tailored to older patients.

## 2. Methods

### 2.1. Animal Procedures

All animal experiments were approved by the animal ethics committee of the Hebrew University of Jerusalem (Institute review board (IRB) approval MD-19-15962-4). Female C57bl mice were used in all the experiments. The young group were mice aged 6–8 weeks, and the adult group were aged 8–10 months on the first day of the experiment. Apical periodontitis was induced as previously described [6]. Briefly, mice were anesthetized with ketamine–medetomidime (concentration of 70 mg/kg ketamine hydrochloride (Vetoquinol, CAS 1867-669) and 0.9 mg/kg medetomidine hydrochloride ((Cepetor, CP-pharma GmbH, CAS 86347-15-1) diluted in PBS), and the pulp of the first mandibular molar (M1) on the right side was exposed in the mesial and distal pulp horns. M1 on the left side was used as a control (the pulp of the right side M2 was also exposed for flow cytometry experiments). The animals received an antisedant ((atipamezole hydrochloride (Eurovet Animal Health, CAS 104075-48-1), concentration of 4 mg/kg, diluted in PBS), at the end of the procedure. During the first three days of the experiment the animals received a daily dosage (0.05 mg/kg on the day of the procedure, and 0.1 mg/kg for another 2 days) of buprenorphine (vetmarket 163451) to relieve pain. During the course of the experiment the animals’ weight was monitored and the animals received soft food daily in order to reduce pain during chewing and to encourage biofilm formation. The teeth were left to become contaminated by the oral flora for 42 days as previously described [6,9]. After this time period, the animals were anesthetized with ketamine (162.5 mg/kg)-xylazine (75 mg/kg, Eurovent Animal Health, CAS 7361-61-7) and euthanized by cervical dislocation. 

For histological sections, mandibular bone blocks including the molars were cut and the soft tissues were peeled off. The bone blocks were rinsed with PBS and left overnight in PFA (paraformaldehyde) 4%, then rinsed thoroughly with phosphate buffered saline (PBS). For histology and micro-CT *n* = 15 (young), 29 (adult). For flow cytometry, see relevant detailed section following.

### 2.2. Micro-CT

Samples were scanned for micro-computed tomography (micro-CT) (µCT 40, Scanco Medical AG, Bassersdorf, Switzerland) using the following parameters: energy of 70 kV, intensity of 114 μA, exposure time of 200 ms, and slice increments of 6 μm in each plane (voxel size of 6 μm^3^). The area of the distal apical constriction (AC) and volume of the distal canal and distal apical bone resorption were calculated using Scanco software. Each scanned tooth was aligned by 3 points, on 2 planes: (1) the mesial apical constriction, (2) the distal apical constriction, (3) the mesial coronal opening of the canal. This gave sagittal slices with a central slice including the pulp chamber, both mesial and distal canals, and the apical foramens which were used for PA lesion analysis. The sagittal aligned samples were flipped to obtain axial slices, which were used for canal volume and apical constriction analyses. For PA lesion volume calculation: the radiolucent area around the distal periapical region was manually delineated in 79 adjacent slices; 39 buccal and 39 lingual to the central slice of each tooth. The volume (3D analysis) of the normal periodontal ligament (PDL) (in control teeth) and the PA lesions (in contaminated teeth) was calculated. In addition, in order to evaluate the accurate area of the inflammatory lesion, we extracted the average volume of the normal PDL area from the PA volume of each contaminated tooth (according to age groups). For apical constriction calculation: the position of the apical constriction of the distal canal was assessed by the sagittal and coronal sections, and the axial slice with the smallest area of the canal in that position was chosen for two-dimensional (2D) analysis. The area of the canal in the chosen section was calculated by the volume of interest (VOI) minus the bone volume (BV). Similarly, the distal canal volume was calculated (3D) by subtracting the BV from the VOI in all the sequential axial slices from the coronal section of the canal (determined by the sagittal slices) until the slice of the apical constriction. To statistically compare the results, a Student’s *t*-test was used.

### 2.3. Histological Slide Preperation

After the CT scan, bone blocks, rinsed with PBS, were placed in EDTA (0.5M, pH 8) for 10 days. EDTA was replaced every 3–4 days. For Paraffin sections (hematoxylin eosin (H&E), Brown and Brenn (B&B), tartrate-resistant acid phosphatase (TRAP) staining) tissues were dehydrated with increasing concentrations of ethanol, then placed in xylene, and in paraffin blocks. Paraffin blocks were sliced to a thickness of 6 microns. For cryosections (immunofluorescent staining): after ethylenediaminetetraacetic acid (EDTA) treatment, tissues were placed in a 30% sucrose solution overnight in 4 °C, and then in Optimal cutting temperature compound (OCT compound) in −80 °C. Cryo-blocks were sliced to a thickness of 10 microns and kept in −20 °C until staining. 

#### 2.3.1. H&E Staining

Hematoxylin eosin (H&E) staining was done as previously described [10]. Slides were deparaffinized (Xylene 10 min twice) and rehydrated with descending concentrations of ethanol to double distilled water (DDW) (100%—5 min, 90 and 70%—2 min each). Thereafter, they were stained with hematoxylin (Sigma-Aldrich, Jerusalem, Israel, MHS 16) for 1 min, rinsed with tap water, stained with eosin (Sigma-Aldrich, Jerusalem, Israel, HT110116) for 30 s and rinsed with tap water. Then, the slides were placed in DDW and dehydrated with increasing concentrations of ethanol (70, 90, and 100%—2 min each), followed by xylene, and covered with a coverslip with entelane.

#### 2.3.2. B&B Staining

Brown and Brenn (B&B) staining was done as previously described [10]. Slides were deparaffinized (xylene (Gadot, CAS 1330-20-7) 10 min twice) and rehydrated with descending concentrations of ethanol (100%—5 min, 90 and 70%—2 min each) to tap water (for 10 min). Thereafter, they were stained with a sequence of the following solutions; Harris haematoxylin (Sigma HHS32)—5 min, acid alcohol (1% HCl in 70% ethanol)—5 s, saturated lithium carbonate (Sigma 62470) in DDW—5 s, Hucker’s solution (0.2% crystal violet (sigma 61135)), 2% ethanol, 0.8% ammonium oxalate ((sigma 221716) in DDW)—2 min, iodine solution (0.33% iodine (BDH CAS 7553-56-2)), 0.66% potassium iodide ((J.T. Baker CAS 7681-11-0) in DDW)—15 s, ether acetone (50% Ether (Daejung CAS 60-29-7)), and 50% acetone (Gadot, CAS 67-64-1)—10 s, basic fuchsin (0.007% basic fuchsin (Sigma 857343, 7.31% ethanol in DDW))—3 min, acetone—10 s, picric acetone (0.1% picric acid in acetone EMS 26105-06)—20 s, acetone–xylene I (33.3% acetone, and 66.6% xylene)—5 s, acetone–xylene II (25% acetone, 75% xylene)—5 s, and xylene—1 min. The slides were mounted with entelane (mercury 1.07961) and covered with a coverslip. For analysis, each canal was divided into three parts, and evaluation was done by manually determining the most apical localization of bacteria in each canal. Statistics were performed using Fisher’s exact test. 

### 2.4. H&E Periapical Computerized Analysis

A computer program was developed for colorimetric evaluation of the cell density of the periapical region on the H&E slides. A python computer program was developed for colorimetric evaluation of the cell density of the periapical region on the H&E slides (the code is available on github via the following link: https://github.com/elishevag16/cell-density-analysis, (accessed on 3 February 2021). The program enables one, for each slide, to calibrate the parameters of HSV (hue, saturation, value) range. The parameters were calibrated for each slide to receive an optimal range in which the nuclei of the cells were maximally included, and the background colors were minimized. Then, the periapical region was cropped, and the computer calculated cell density as a percentage of the included pixels from all pixels (in the cropped area). The output was used for comparison between the different experimental groups as a proxy for cell density in area of interest. The technique was validated by showing a significant difference between treated and control teeth, and by showing a correlation with the micro-CT results.

### 2.5. Tartrate-Resistant Acid Phosphatase (TRAP)

Paraffin slides were stained for osteoclasts with TRAP staining, as previously described [11]. Slides were deparaffinized (xylene 2 min × 2 (and rehydrated with descending concentrations of ethanol (100% × 3, 90%, 70%—2 min each) to tap water. Tissues were marked with a pap pen, and covered with TRAP incubation solution (50 mL TRAP buffer: sodium acetate (J.T.Baker CAS 127-09-3) 16.4 g/L and sodium tartarate (Sigma CAS 6106-24-7) 11.6 g/L in DDW, titrated to pH 5 with 2.5 mL AS-BI substrate mix: 6 g/L naphthol AS-BI phosphate sodium salt (Sigma N2250), 10% N’N-dimethylformamide (Sigma 227056), 70% methanol (Gadot CAS 67-56-1) and 20% DDW, mixed with 0.025 g Fast red TR salt (Sigma CAS 89453-69-0). The slides were incubated for 30 min at 37 degrees Celsius until red staining was detected under a microscope. The slides were washed with DDW, counterstained with hematoxylin for 30 s, washed with tap water and covered with a coverslip with hydromount 17,966 (EMS).

For analysis, the periapical bone border length was measured using ImageJ software. The number of TRAP-positive multinucleated (≥3 nuclei) cells was counted manually around the necrotic canals, and the number of osteoclasts per mm on the bone border was calculated as previously described [9,12]

### 2.6. Immunofluorescent Staining

Cryosections were stained with lymphocyte antigen 6 complex locus G6D (Ly6G) primary antibody, and a conjugate fluorescent secondary antibody, as previously described [13]. Cryosections were defrosted at room temperature for 1 h, followed by 10 min at 37 degrees Celsius. The remaining OCT was rinsed with PBS. Tissues were marked with P pap pen, and incubated at room temperature in a wet chamber with blocking solution (5% FCS (biological industries, Beit Haemek)) and 1% Triton ((Sigma-Aldrich, Jerusalem, Israel, CAS 9002-93-1) in pbs) for 1 h. Thereafter, tissues were incubated overnight at 4 °C in a wet chamber with the primary antibody vs. Ly6G (BD biosciences, 551459) diluted at a ratio of 1:100 in blocking solution. PBS rinses were performed, and the tissues were incubated for 2 h at room temperature in a wet chamber with the secondary antibody (Invitrogen A11077) diluted at a ratio of 1:200 in blocking solution. Slides were rinsed, stained with 5 µM DAPI (abcam AB-ab228549) at room temperature for 10 min, rinsed, and covered with a coverslip with mounting medium (VECTASHIELD^®^ Mounting Medium (H-1000, Zotal)). Images were obtained with NIKON-Ti X40 magnification (N.A. 0.75 dry), using Photometrics Prime 95B camera.

### 2.7. Flow Cytometry

The cells were isolated from the periapical region of the first mandibular molars as previously described [14]. After the animals had been euthanized, jaws were extracted, and the soft tissues were peeled off. Under magnification, the first and second mandibular molars were extracted, and cleaned from remnants of bone and gingiva. The tissue was placed in working solution (2% FCS in PBS) with DNAse I (Sigma DN25) 1 mg/mL and Collagenase II (Worthington LS004177). Each tube consisted of a pool of 3–5 mice, and each group was run 3 times on sequential days (*n* = 10 (young), 15 (Adult)). The solution with the teeth was incubated in a shaker at 37 °C for 25 min, and EDTA (0.5 mM, pH 8) was added (at a concentration of 1%) and incubated for another 10 min at the same conditions. The solution was filtered (with 70 micron filters), centrifuged (4 °C, 1000 rpm, 5 min), upper liquid was removed, and cells were stained with an antibody mix (Pacific Blue™ anti-mouse CD45.2 antibody (109820), PE/Cy7 anti-mouse/human CD11b antibody (101216), Brilliant Violet 711™ anti-mouse Ly-6C Antibody (128037), APC anti-mouse Ly-6G Antibody (127614), PE anti-mouse CD3 Antibody (100206), PE anti-mouse CD3 Antibody (100206), all from Biolegend, diluted 1:250 for each antibody in working solution) for 20 min on ice, rinsed, and fixated with cell preservative (Streck 213350) at a ratio of 1:6. The stained samples were run in the BD LSRFortessa™ cell analyzer (BD Biosciences, US) flow cytometer and analyzed using FlowJo software (Tree Star, BD Biosciences, US). 

### 2.8. Statistical Analysis

H&E radicular pulp diagnosis, and B&B staining were analyzed by Fisher’s test (online calculator: https://www.graphpad.com/quickcalcs/contingency1/ (accessed on 3 February 2021)). Micro-CT, TRAP, and H&E computerized results were analyzed by Student’s *t*-test (using excel software). The difference in the variance was tested by F-test (online calculator: https://www.statskingdom.com/220VarF2.html (accessed on 3 February 2021)).

## 3. Results

### 3.1. Radicular Pulp Reaction

**Figure 1:**
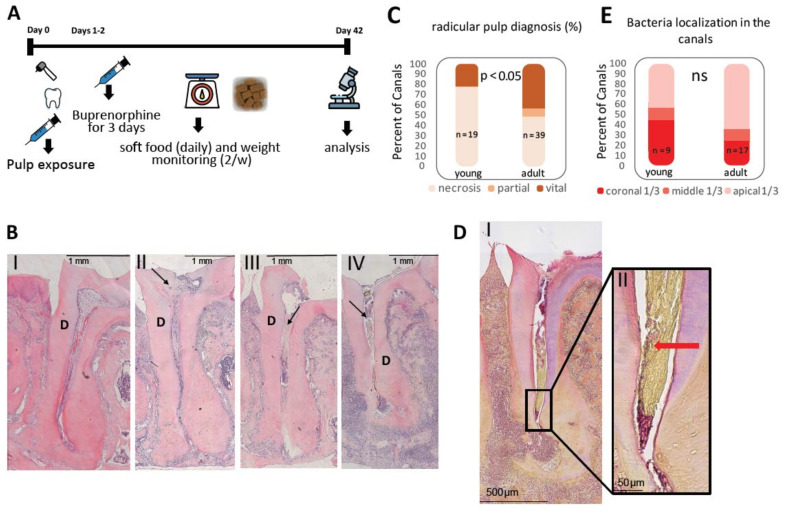
Experimental setup. (**A**) Scheme of experiment on a time scale. The experiments began with mesial and distal pulp exposure on the 1st mandibular molar unilaterally. The contralateral tooth was left as a control. To reduce pain, buprenorphine was injected to the animals for 3 days. The animals were left for 42 days, to develop the periapical lesion. During this time-course the animals received soft food daily and were monitored for weight loss. After 42 days, the animals were euthanized, and their jaws and teeth were removed and processed for analysis (**B**) H&E representative images of the different histological diagnosis in the root canals. D = dentin. I. control tooth with normal pulp diagnosis. II. tooth 42 days after pulp exposure presenting canal obliteration (arrow) and vital pulp. III. Partial necrotic canal. Coronal half of the canal presents necrosis (arrow), while apical half presents vital pulp. IV. Necrotic canal (arrow) (**C**) pulp diagnosis (%). percent of the canals after pulp exposure with the different pulp diagnosis, comparing the young and adult groups. *p* value < 0.05 by fisher test, comparing vital pulp to partial or necrotic pulp (*n* = 19 (young), 39 (adult)). (**D**) Representative Brown and Brenn image of bacteria in the apical 1/3 of an infected canal (I). The area enclosed in the black rectangle is an enlarged in image (II) where the bacterial biofilm can be seen as small blue dots near the canal wall (red arrow). (**E**) Localization of bacteria in the canals. The bar graph presents the percent of canals (out of all the canals excluding canals with vital pulp) with bacteria in the different locations of the canal. *p* value = 0.41 (fisher test), comparing the coronal 1/3 to the apical and middle 1/3 (*n* = 9 (young), 17 (adult)).

The aim of this study was to investigate the effect of age on the development of AP. We chose an established mouse model for induced AP [6,7,8]. In our experiments we compared two age groups of mice: young mice (6–8 weeks), and mid-age mice (termed adults: 8–10 months) as a model for the difference in development of AP in adolescence and adult age. All results for the different analyses per mouse are presented in Supplementary Table S1. The outline of the experiment is presented in Figure 1a.

We determined the outcome of pulp exposure by histological diagnosis (Figure 1B,C). In the control group (non-treated teeth) all histological samples (*n* = 45/45) presented normal and vital radicular pulp (see Figure 1BI). In the experimental group, most of the young mice canals and a large part of the adult canals presented necrotic pulp (Figure 1BIV) (*n* = 15/19 young, 19/39 adult), although some of the canals remained vital or partially vital following 42 days of pulp exposure (Figure 1BII, III). In most cases the vital canals presented canal obliteration (*n* = 4/4 young, 8/11 adult) (Supplementary Figure S1). All necrotic canals were patent (assessed by micro-CT, results not shown).

We quantified the percentage of each canal diagnosis for the different age groups (Figure 1C). From the statistical analysis of these results, we concluded that the adult group had significantly more canals that presented vital pulp after 42 days of pulp exposure compared to the young group. This phenomenon may be explained by the formation of a mechanical barrier due to denser connective tissue that characterizes the pulp tissue of older teeth [15], or due to immunological changes (see discussion). For this study exploring apical periodontitis, all the samples that exhibited vital pulp were excluded from subsequent analyses.

Next, we proceeded to examine and compare bacterial localization in the canals between the two age groups by B&B staining (Figure 1D–E). As presented in Figure 1E, there was no statistically significant difference in bacterial localization between the two age groups. This result indicates that in cases where necrosis was evident, the bacterial stimulus (in terms of bacterial localization) was similar for both age groups.

### 3.2. Age-Related Mechanical Barrier

**Figure 2:**
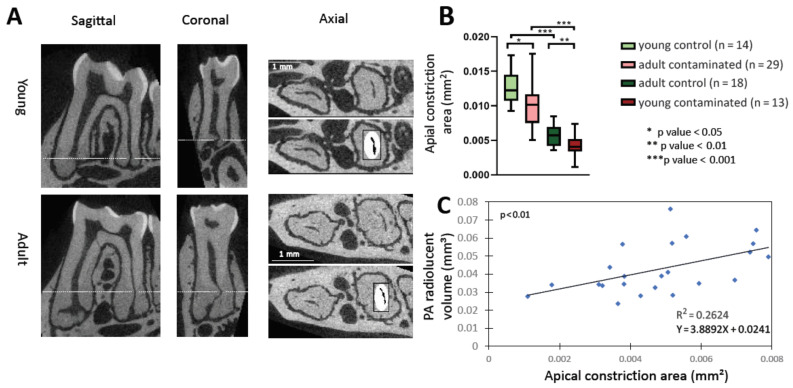
Apical constriction. (**A**) Representative micro-computerized-tomography (micro-CT) images of apical constriction measurement. Sagittal and coronal slices were used to assess the apical constriction location, which was then measured accurately on the chosen axial slice. (**B**) Apical constriction area for control and contaminated teeth of each age group (*n* = 14 (young control), 13 (young contaminated), 29 (adult control), 18 (adult contaminated)) * *p* value < 0.05, ** *p* value < 0.01, *** *p* values < 0.001. (**C**) Linear correlation between the apical constriction area and the lesion volume (see Figure 5) for contaminated teeth (young and adult combined) up to 0.08 mm^2^ (above this value the graph reaches a plateau—see Supplementary Figure S3).

For the formation of AP, it is necessary for canal bacteria or their products to irritate the periapical (PA) tissues. In order to do so they must pass through the canal and the apical constriction (AC). Therefore, we measured the size of the canals and the AC by micro-CT and compared the different age and experimental groups (Figure 2). The volumes of the young mice root canals were larger than those of adult mice (Supplementary Figure S2), as previously established [16]. Figure 2A demonstrates the method we used to measure the area of the AC. The distance between the apical constriction and the major apical foramina is larger in adult compared to young mice, as previously established [17]. Figure 2B presents the size of the AC for the different groups. The young groups present a significantly larger AC area compared to the adult groups. Moreover, for each age group the contaminated teeth presented significantly smaller AC areas compared to the control teeth. This phenomenon may be explained by a reaction of the pulp to the bacterial stimuli causing reactive dentin formation which narrowed the size of the AC lumen [18,19]. Next, we checked whether there was a correlation between the size of the canal volume (Supplementary Figure S2) or the AC (Figure 2C and Supplementary Figure S3) in the contaminated teeth and the size of the lesion (as measured by micro-CT—see Figure 5). We found that there was a significant positive correlation between the size of the canal volume or AC and the size of the lesion in each mouse up to a certain size (for the AC ~0.09 mm^2^), and thereafter, it reaches a plateau (see discussion), as was deduced from the decrease in the graph slope (see Supplementary Figures S2 and S3). We conclude from this analysis that an age-related mechanical barrier could be a significant factor in the determination of AP progression.

### 3.3. Apical Tissue Inflammatory Reaction

**Figure 3:**
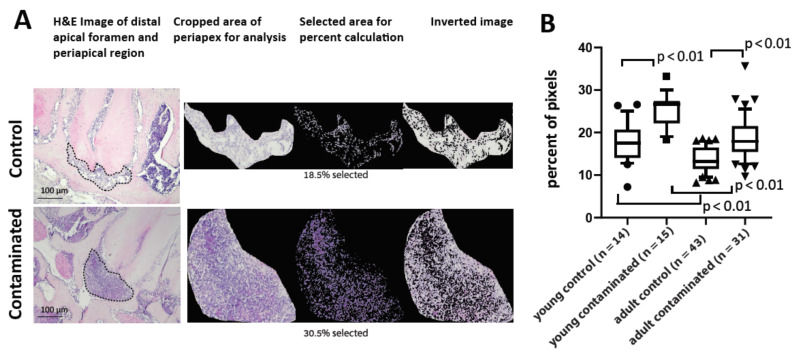
Computerized analysis of periapical cell density. We developed a computerized test to quantify the percentage of nuclei in the PA region to evaluate the cell density. (**A**) Representative images of the analysis method. (Left) representative images of a control tooth (with normal PDL region in the apex), and a contaminated tooth (with an immune cell infiltrate in the apical region). The dashed line delimits the cropped area of the periapical region for analysis. (Right) 3 images from the computer’s output (from left to right): the cropped area, the cell nuclei selected for quantification, and the inverted image, indicating that, indeed, all the cells were included in the analysis. The percentage of the image stained (representing the number of nuclei, indicating cell density) is presented for each tooth. (**B**) Quantification of cell density. The Y axis shows the percentage of pixels (from the computer output), which represents cell density. The cell density of the control and contaminated groups of both young and adult mice are presented. n (number of canals): young control = 21, young contaminated = 15, adult control = 43, adult contaminated = 31.

We then proceeded to examine the effects of periapical inflammation in both age groups. The H&E stained slides of the necrotic canals presented a significantly larger periapical area compared to the controls (see micro-CT results in Figure 5 for quantification of the bone resorption volume). The periapical space in the contaminated teeth was characterized by the presence of dense connective tissue and inflammatory infiltrate of mononuclear cells and PMNs, advanced bone resorption and cementum resorption lacunas adjacent to the apical lesion. This histological picture was severe in 75% of the necrotic canals in the young group (*n* = 9/12), and 59% of the necrotic canals in the adult group (*n* = 10/17). To accurately quantify the density of the cellular infiltration in this region, we developed a colorimetric computer algorithm. Figure 3A presents an example of the ability of this method to quantify the difference between a control and a contaminated tooth. Figure 3B compares the different age groups for the control and contaminated periapical (PA) regions. The results show that the contaminated teeth in both age groups presented significantly higher cell density than the control teeth, indicating that we can accurately compare the cell density in the PA region. Moreover, we found a positive correlation between the results in this method and the micro-CT results for bone resorption for each tooth (see below Figure 5 and Supplementary Figure S4), strengthening the validity of this technique. When comparing control teeth for both age groups, young mice presented significantly higher cell densities (in the intact PDL tissue) compared to the adult mice (see Discussion). Finally, in the PA region of the contaminated teeth, young mice presented a significantly higher cell density on average compared to the adult mice, indicating a more intense cellular infiltration in the periapex in reaction to bacterial stimuli. Another observation is the difference in the variance between the young and adult contaminated groups. The variance of the adult group cellular infiltration is significantly higher than the young group (*p* value = 0.003 using F test), and some individual teeth presented very high cell density in PA tissue (see Supplementary Table S1).

### 3.4. Cellular Composition of the Lesion

**Figure 4:**
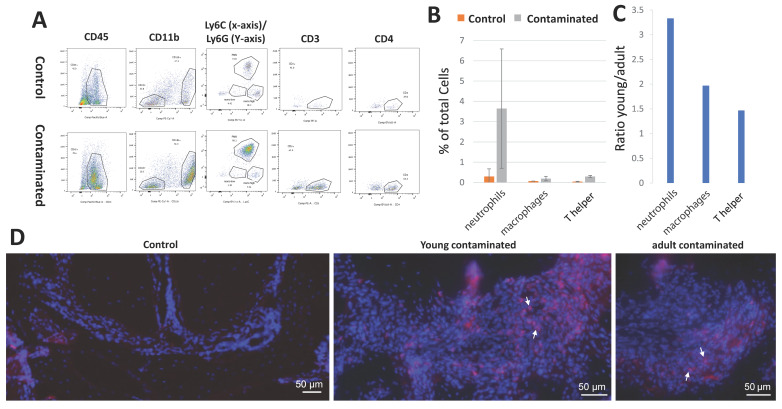
Cellular composition of the lesions. Cells were extracted from the periapical region, stained for immune markers, and analyzed by flow cytometry. (**A**) Representative gating for a panel of immune cells in the control and contaminated teeth; total cells were gated for CD45 (representing all immune cells), CD45+ cells were gated for CD11b+ (myeloid cells) and CD11b- (lymphoid cells). CD11b+ cells were gated for Ly6C (macrophages), and Ly6G (neutrophils). CD11b- cells were gated for CD3 (T lymphocytes). CD3+ cells were gated for CD4 (T helper cells). (**B**) Percentage of the different immune cell types comparing control to contaminated teeth in the young group. (**C**) Ratio of the immune cells between the young and adult contaminated teeth. (**D**) Immunofluorescent staining for Ly6G of the periapical regions of control (young), and contaminated (young and adult teeth). White arrows point to stained cells (neutrophils).

We further investigated immune cell composition of the lesions by flow cytometry. We isolated cells from normal PDL (in controls) and from PA lesions (in contaminated teeth), stained them with a panel of immune cell markers, and quantified the relative abundance of different cell populations. Figure 4A demonstrates representative gating for our results. Cells were gated for the markers: CD45, the CD45+ cells were next gated for the myeloid CD11b marker. CD11b+ myeloid cells were further gated for Ly6G and Ly6C, defining populations of neutrophils and macrophages, respectively. The CD11b-, lymphoid cells, were gated for CD3 to identify T-cells, and the CD3+ cells were gated for CD4 to identify the sub-type CD4+ T-helper cells. 

Figure 4A demonstrates an increase in all immune cell types in contaminated teeth compared to the control PDL (periodontal ligament), validating our technique of cell extraction. First, we established the percentage of the different immune cell populations in the young mice as a baseline (Figure 4B). The cells with the highest elevation in the lesion compared to the controls were the neutrophils (Ly6G+), suggesting an important role for this cell type in AP in mice. Next, we calculated the ratio of the different cell types between the young and adult groups (Figure 4C). These results are well correlated to our previous results, i.e., the young group presented higher percentage of immune cells compared to adults. The full flow cytometry results for all groups are displayed in Supplementary Figure S5.

To validate our flow cytometry results, we preformed immunofluorescent staining for Ly6G (Figure 4D). Representative images demonstrate the presence of neutrophils in the PA lesion in young and adult mice, but not in the control teeth. This result validates that immune cells isolated by flow cytometry were indeed the cells from the PA lesion.

### 3.5. Bone Resorption

**Figure 5:**
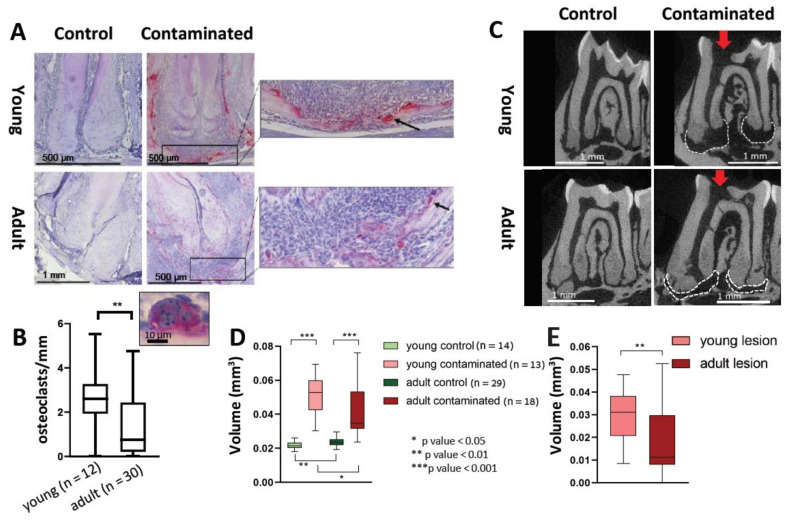
Bone resorption analysis. (**A**,**B**). Tartrate-resistant acid phosphatase (TRAP) analysis. The slides were stained for TRAP, and the number of TRAP positive multinucleated cells per mm of bone border length in the distal periapical region was quantified and compared for the different age groups. (**A**) Representative images. Representative images of control and contaminated teeth for both age groups. The arrows point to TRAP positive cells (stained red). (**B**) Quantification of osteoclasts. The number of TRAP-positive multinucleated cells (≥3 nuclei) per mm on the bone border was quantified and compared between the two age groups (only for the contaminated teeth, *n* = 12 (young), 30 (adult)). (**C**–**E**) micro-CT analysis. Samples were scanned by micro-CT in order to quantify the volume of the periapical lesion. (**C**) Representative images. Representative images of control and contaminated teeth for both age groups. Arrows point to the pulp exposure site. The white dashed line delineates the area of the lesion. (**D**) Volume of the radiolucent area. For all groups the periapical radiolucent volume was calculated. (**E**) Volume of the lesions. The volume of the periodontal ligament (PDL) area of the control teeth was subtracted from the radiolucent area of the contaminated teeth for each age group. The result is the volume of the bone resorption area presented.

One of the hallmarks of apical periodontitis is bone resorption [20]. In order to evaluate bone resorption in the PA region, we preformed histological TRAP staining (Figure 5A,B) and micro-CT evaluation (Figure 5C–E). The osteoclast differentiation process includes the formation of a syncytium, and it was shown that this multinucleation of the cells improves bone resorption efficiency [21]. Our TRAP results revealed a negligible number of osteoclasts in the PA bone border of control slides in both age groups (results are not presented). Within the contaminated teeth, there was no significant difference in the total number of TRAP-positive cells between the groups (young: 6.97 cells/mm, adult: 7.07 cells/mm. *p* value = 0.14). The young mice presented a significantly higher number of multinucleated TRAP-positive cells (*p* value = 0.009).

We quantified osteoclast-mediated bone resorption in the lesion by micro-CT. Our results (Figure 5D) show a significant difference for both age groups between the contaminated and control teeth. Variance was smaller in the control groups (where the apical PDL volume was measured) compared to the contaminated teeth. A significant difference was observed between the control age groups. In these groups the adults presented a significantly higher volume of PDL region compared to the young. This is possibly due to cementum deposition occurring with age [22] causing an increase in the mesio-distal aspect of the adult teeth in the apical area. Buco-lingual cementum deposition does not affect our result because we analyzed the same number of slices for all teeth on the buco-lingual aspect. When comparing the contaminated teeth for both age groups, young mice presented significantly larger PA lesions compared to the adult group. In order to calculate the radiolucent volume as a result of the lesion itself, we calculated the difference between the volume of the lesion in the contaminated teeth and the average volume of the PDL in the control teeth for each age group. The results presented in Figure 5E demonstrate significantly larger bone resorption for the young mice compared to the adult group.

## 4. Discussion

The aim of our work was to study the effect of age on AP development. The experimental design used an established mouse model for induced AP [7,8] and compared two age groups termed young and adult. Surprisingly, only ~50% of adult mice canals, and ~80% of young mice canals presented necrotic pulp (Figure 1C)—a prerequisite condition for AP. In most of the vital canals, pulp obliteration was observed (Supplementary Figure S1), as previously reported [18,19], possibly decelerating the bacterial attack. The reason why adults presented fewer necrotic canals (compared to the young mice) was not tested in this work. One possible explanation could be age-related changes in the pulp resulting in a denser connective tissue [15] that serves as a physical barrier for bacteria. Another biological explanation could be an immunological phenotype related to immune changes that occurs with age [3], which correlates with the other results reported in this field of research. Regardless of the mechanism for this phenomenon, we discarded the vital canals in the following analyses which were aimed to study AP because they are not relevant for this examination. 

Identification of bacteria in the tissue via B&B staining revealed no significant difference in the localization of bacteria in the canal between the age groups (Figure 1E). These results show that the following AP age-related differences occur due to processes that are mediated by the host, rather than environmental (bacteria stimuli) processes. In this context, it is important to emphasize that B&B staining enabled us to identify the localization but not the quantity and composition of the canal bacteria, which can be evaluated by 16S. A differential age dependent microbiome [23,24,25] could further contribute to the difference in AP progression between the age groups and should be further investigated.

The common cause of AP is bacteria or bacterial by products stimuli of the PA tissues [2], which requires them to go through root canals and the AC. We, therefore, measured the size of the canals and AC in the different age and experimental groups (Supplementary Figure S2 and Figure 2). The adult group presented a significantly smaller canal volume and AC area compared to the young group (Supplementary Figure S2A and Figure 2B), most likely due to canal calcification occurring with age, as previously described [16,17]. There was a significant positive correlation between AC area and canal volume with the size of lesion, up to a canal volume of ~0.01 mm^3^ and AC size of ~0.009 mm^2^, and thereafter, the graph reached a plateau (Supplementary Figures S2 and S3). Our interpretation of these results is that the canal volume and the AC area effects the influx of bacteria up to a certain point, when the size is no longer a limiting factor. Overall, according to our results, an age-related decrease in canal and AC size may be one of the causes of less severe AP that occurs with age. Although this observation may obscure the source of the attenuated inflammatory response in the adult mice (mechanical obstruction vs. immune system deterioration), it is important to point out that this mechanical effect also occurs in humans with age, and therefore, should be considered clinically. Moreover, it is possible that age-related canal calcification occurs as a result of an inflamm-aging process. 

We developed a computer algorithm to quantify and compare the cell density in the PA region between the age groups (Figure 3). The intact control PDL presented a higher cell density in the young mice compared to the adults, as previously established [26]. The average cell density in the AP area of the contaminated teeth was significantly higher in the young compared to the adult group, meaning that the young mice had more cells in the PA region to initiate inflammatory bone resorption. Another interesting observation in this experiment, as mentioned, is that the adults presented significantly higher levels of variance, a well-known phenomenon related to age, explained by cumulative environmental influences such as bacterial exposure [27,28]. 

In the pathophysiology of AP, osteoclasts are recruited and differentiated in a cascade caused by inflammatory cells and cytokines such as TNF-α [29,30]. During the maturation process of the osteoclast, it becomes a syncytium, a process which improves its bone resorption capability [21]. We did not find a difference in the total number of TRAP-positive cells in the PA lesions between the young and adult mice. Nevertheless, our results revealed an increase in the number of multinucleated TRAP-positive cells (differentiated cells, as opposed to the mono-nucleated pre-osteoclasts) in the young mice compared to the adults (Figure 5A,B). This result conveys an effect of age on the process of osteoclast differentiation, resulting in a reduced efficiency of bone resorption occurring with age. This phenomenon is in line with other studies showing an age-related deterioration in the differentiation capacity of stem cells, rendering them less prone to differentiation [31]. Indeed, our micro-CT results (Figure 5C–E) indicate reduced bone resorption in the lesion for the adult mice compared to the young. 

Overall, our results—using several different methods—conclude that, on average, adult mice present lower levels of inflammatory parameters; necrosis, cell density of the periapical infiltrate, and alveolar bone resorption compared to young mice. We suggest that part of the reason for this phenomenon is related to reduced immune functioning that occurs with age [32]. In a seemingly contradictory manner, the adult mice with the attenuated immune response reacted to the canal infection in a way that presented less tissue damage. Our interpretation of these results is that AP presents a unique phenomenon, as bacteria stimulate the immune system from a remote position (inside the canal or even in the dental tubuli) and are not in direct contact with the immune cells [2]. Therefore, in contrast to other infectious diseases—where a strong immune system can eradicate the pathogen and overcome the infection—in AP, the disease is not self-healing, and eventually the immune system itself will cause damage. 

To conclude, this study presents a model for studying the effect of age on AP in mice. We showed that age is positively correlated with a reduced inflammatory reaction and bone resorption, which may be due to both an age-related mechanical barrier and reduced immunity. Therefore, we suggest that patients’ age should perhaps be considered clinically for follow-up time after treatment, or for decision making about secondary treatment in asymptomatic teeth with a radiographic lesion. Moreover, we showed that neutrophils are a major player in AP in mice and are most significantly reduced with age. Clinical studies should be designed to investigate whether or not there is a similar age effect on AP progression in humans.

## Data Availability

The detailed results per mouse presented in this study are available in Table S1. Further details are obtainable upon request from the corresponding author. The software in this study was deposited in GitHub (see Methods) and is publically available.

## Supplementary Materials

The following are available online at https://www.mdpi.com/2073-4409/10/3/671/s1, Figure S1: Canal Obliteration, Figure S2: Canal volume, Figure S3: Correlation between apical constriction area and PA lesion volume, Figure S4: Correlation between the H&E computerized analysis technique and the micro-CT results, Figure S5: Flow cytometry, Table S1: Results per mouse.
