# Supplementary figures and images for: A Mouse Model for Studying the Development of Apical Periodontitis with Age
Source: Cells. 2021 Mar 17;10(3):671. doi: 10.3390/cells10030671 (PMC8002842; doi:10.3390/cells10030671)

**A**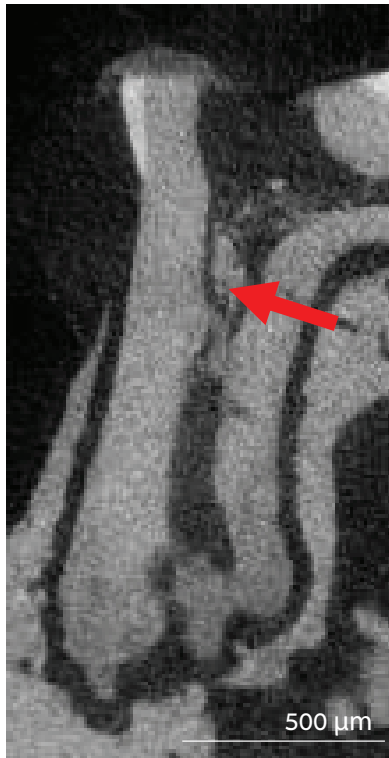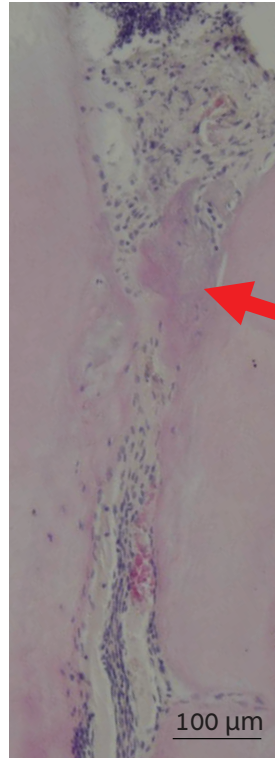**B**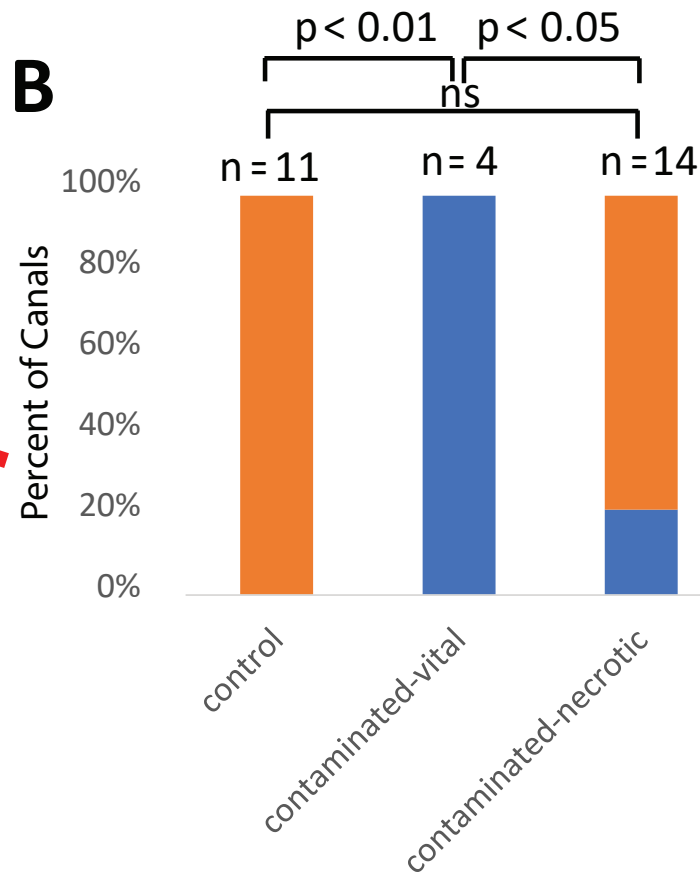**C**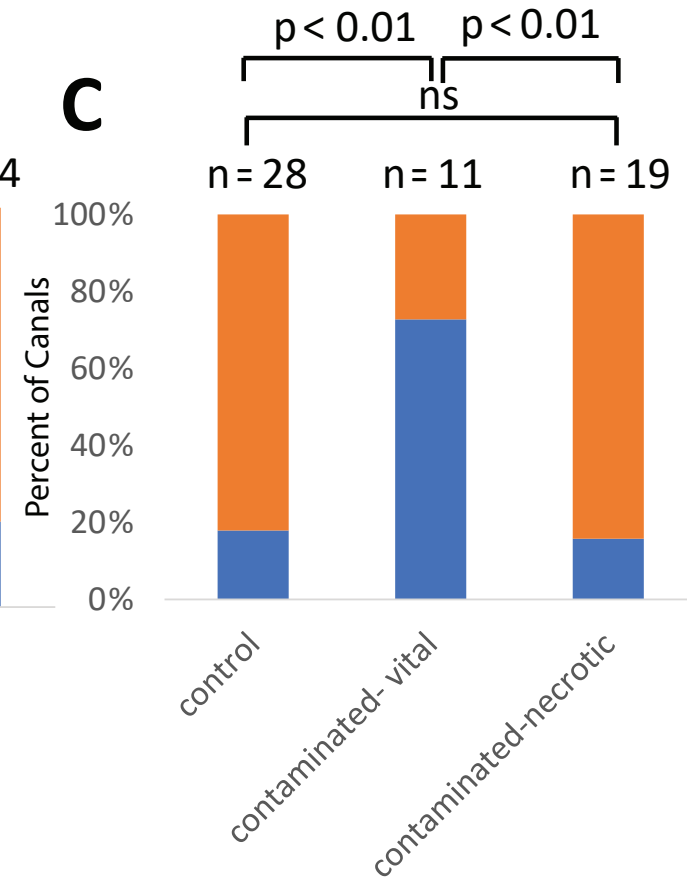

■ canal obliteration ■ no canal obliteration

Young

Adult

Supplement: Supplementary file 1 [file cells-10-00671-s001.zip › supplementary figure 1.pdf]

A

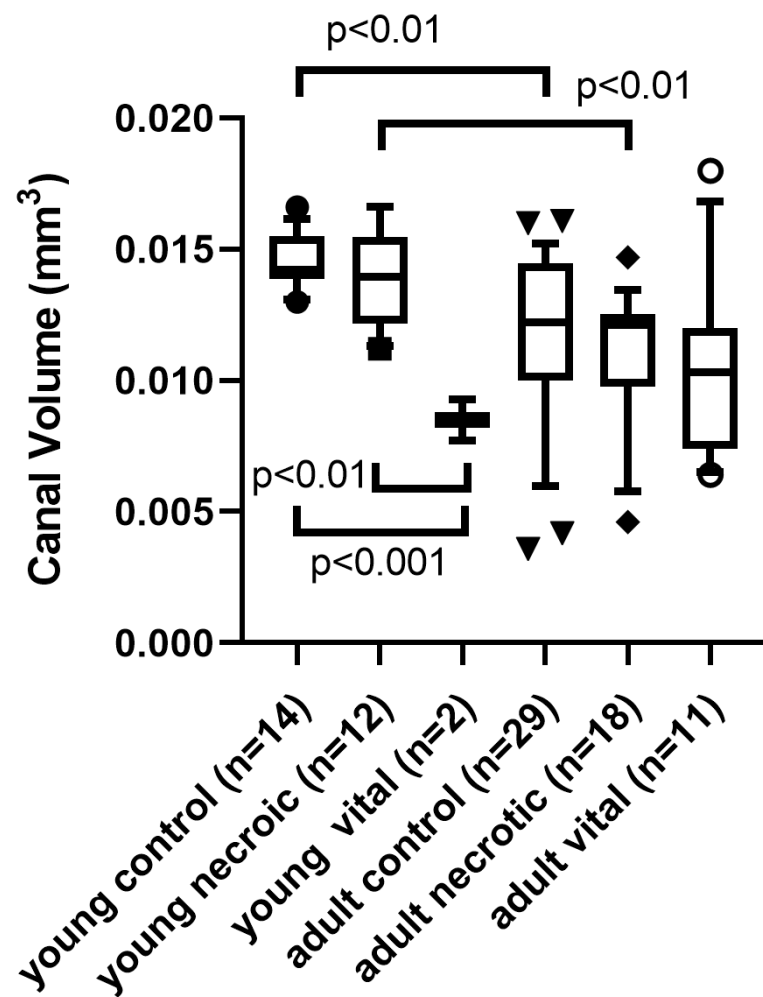

B

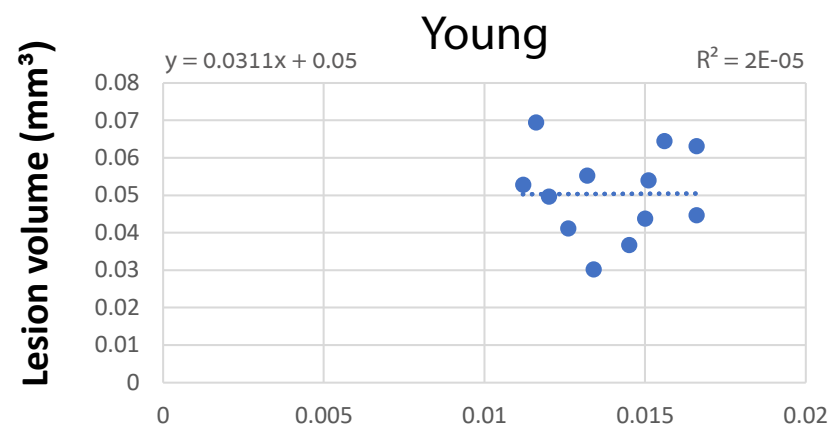

C

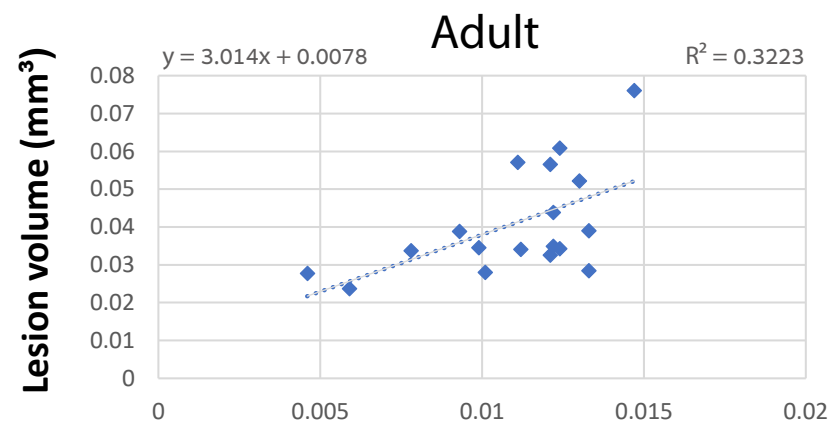

D

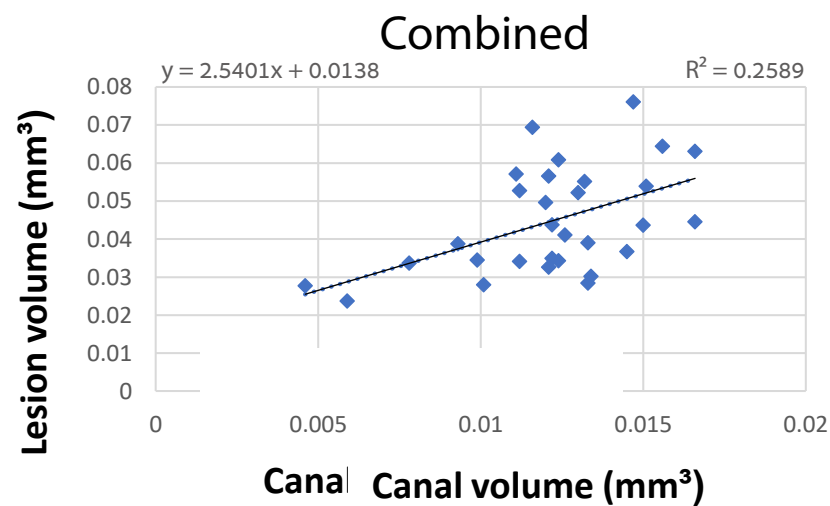

Supplement: Supplementary file 1 [file cells-10-00671-s001.zip › supplementary figure 2.pdf]

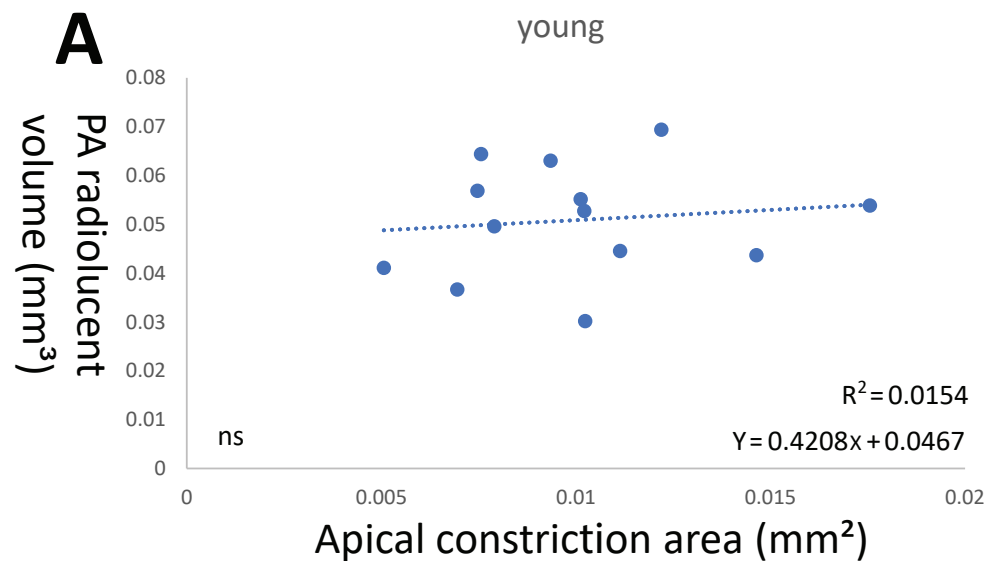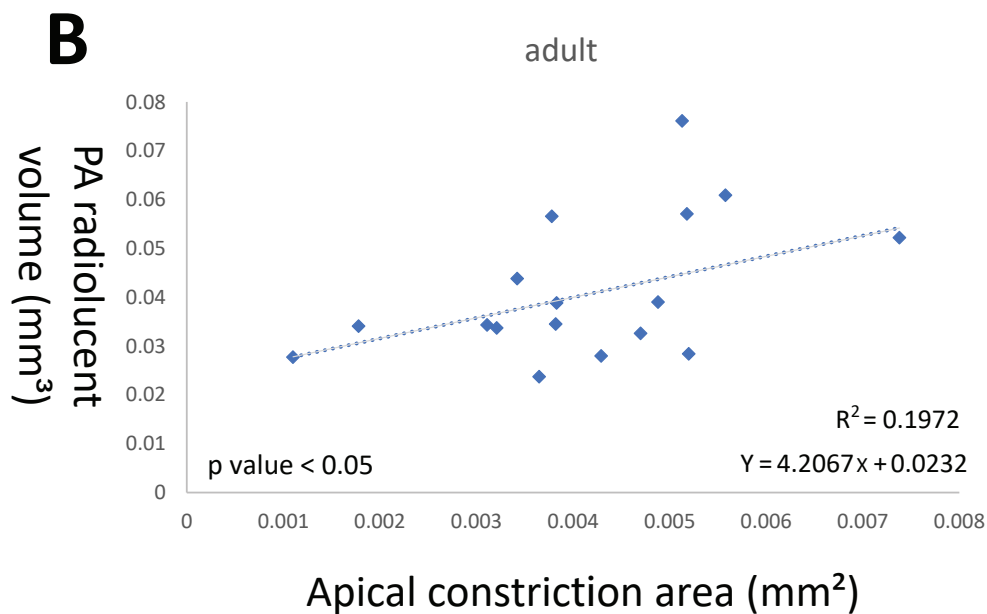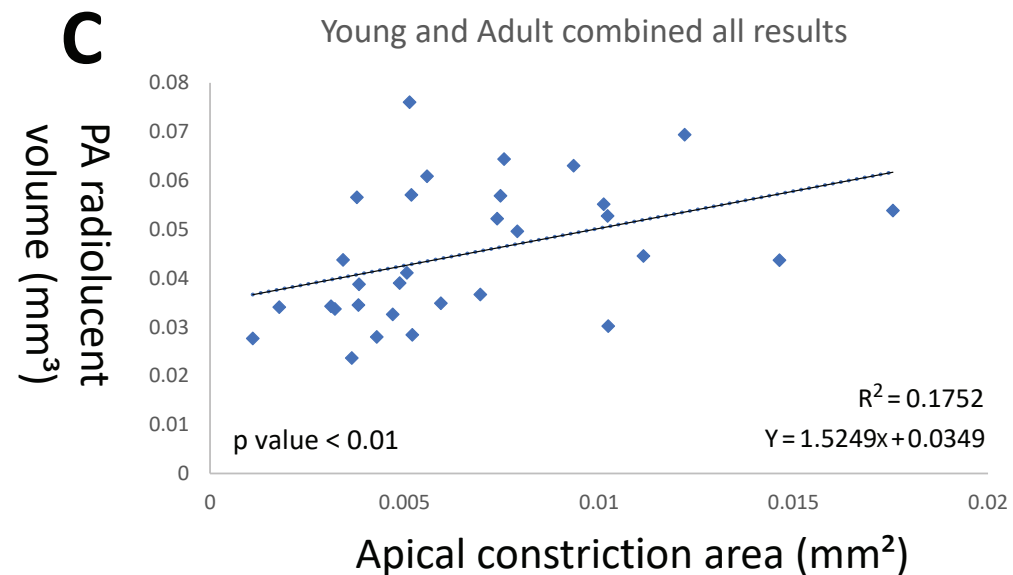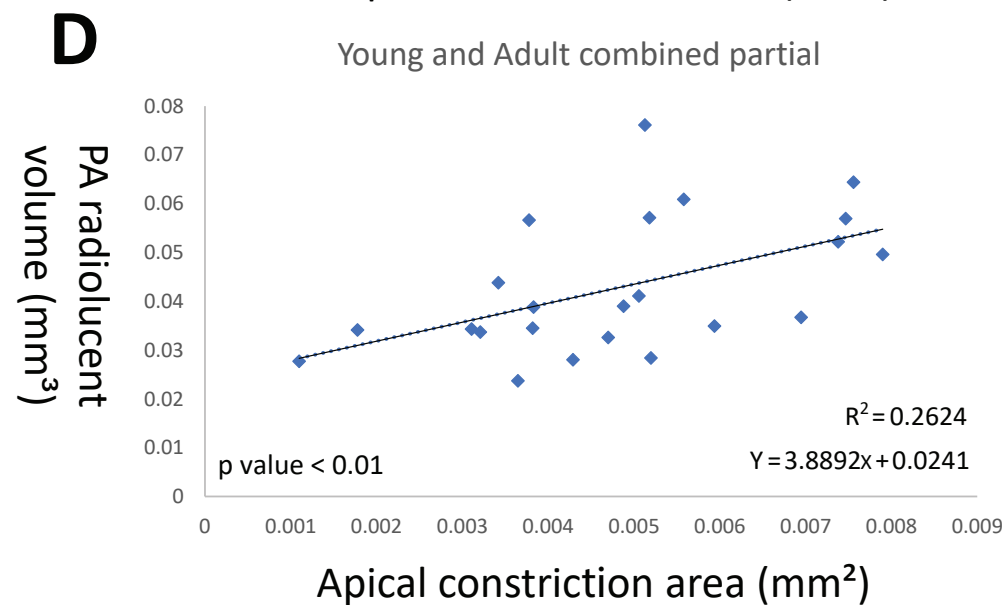

Supplement: Supplementary file 1 [file cells-10-00671-s001.zip › supplementary figure 3.pdf]

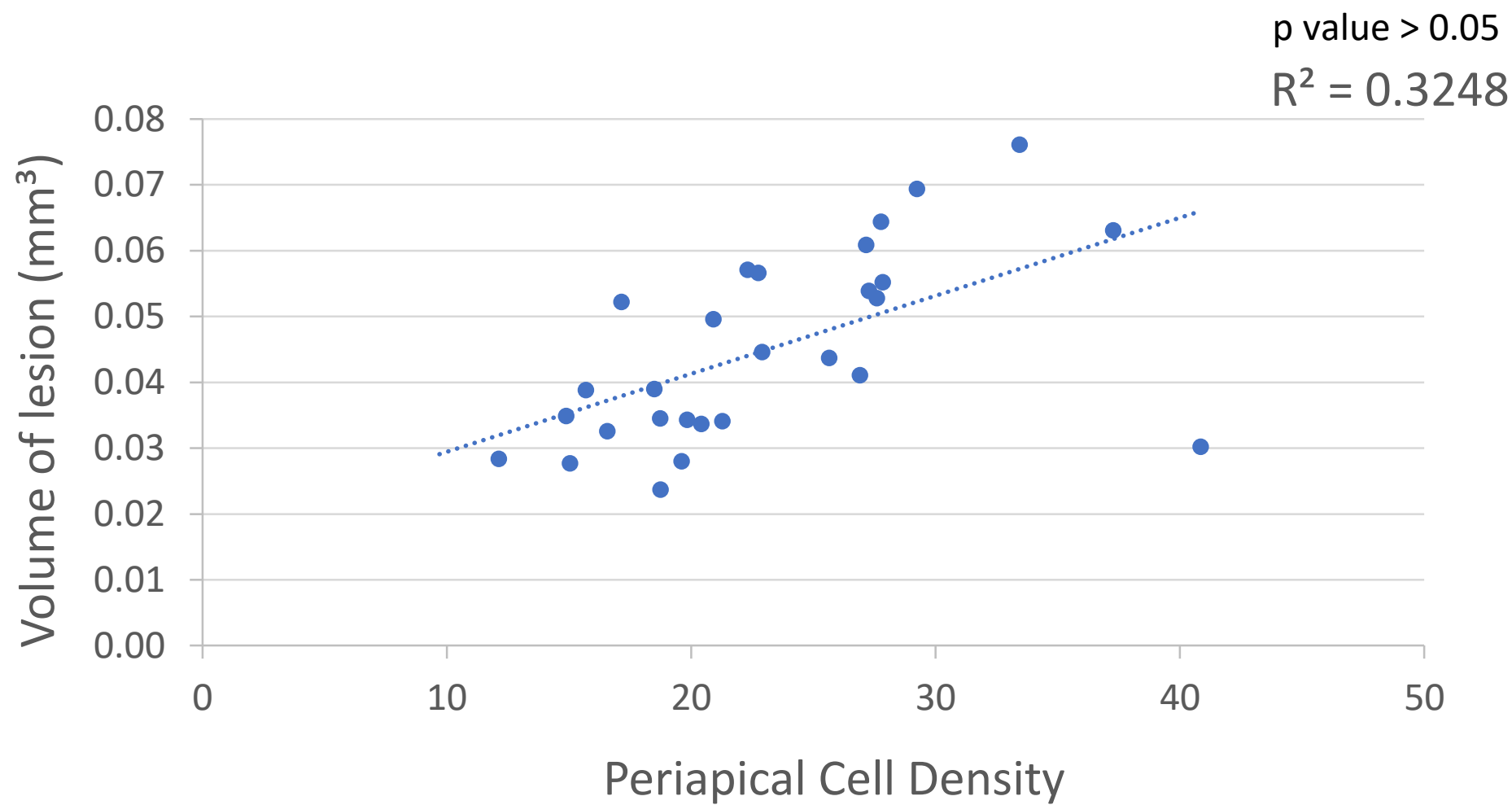

Supplement: Supplementary file 1 [file cells-10-00671-s001.zip › supplementary figure 4.pdf]

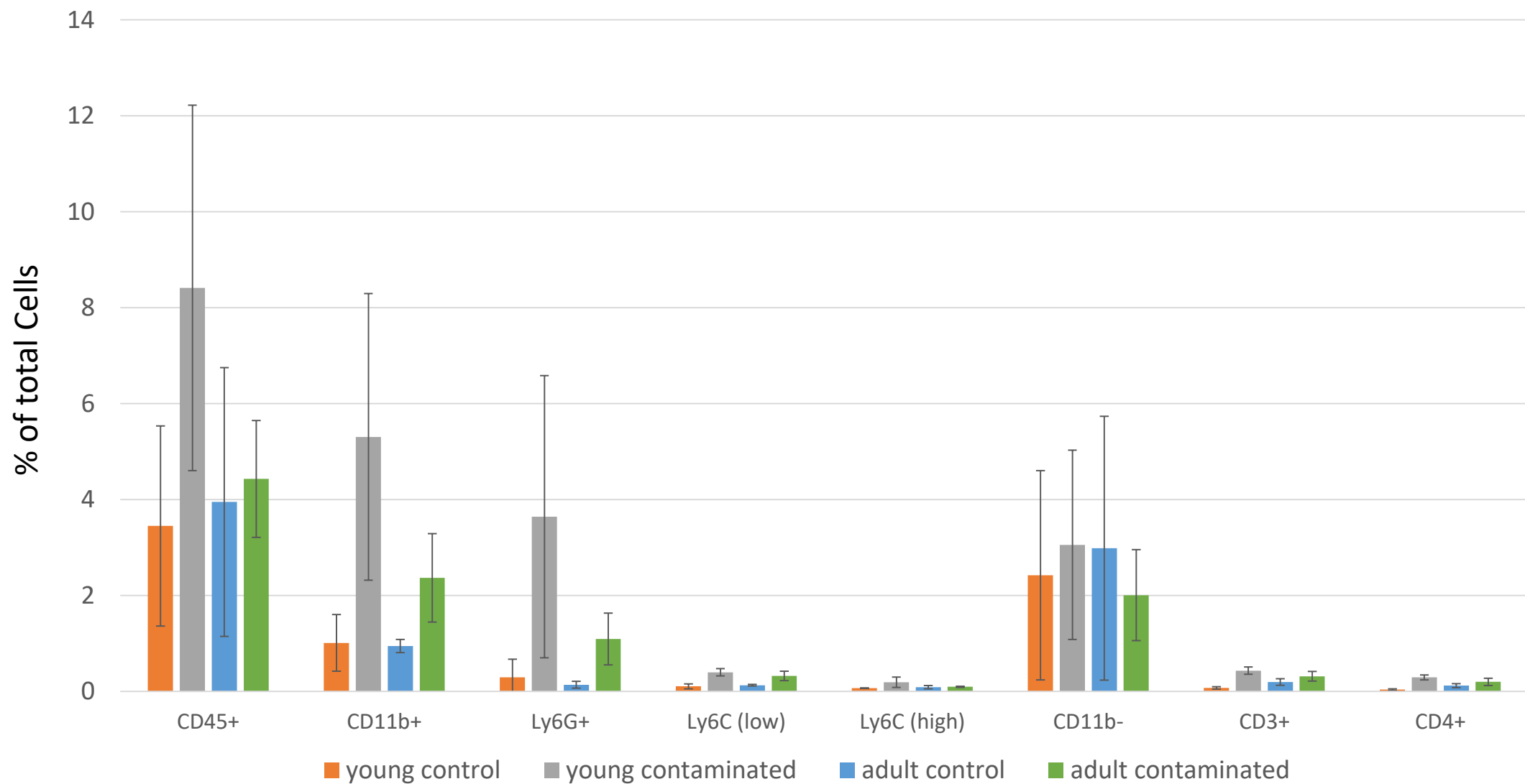

Supplement: Supplementary file 1 [file cells-10-00671-s001.zip › supplementary figure 5.pdf]
